# Supplementary material for: Leucoverdazyls as Novel Potent Inhibitors of Enterovirus Replication
Source: Pathogens. 2024 May 15;13(5):410. doi: 10.3390/pathogens13050410 (PMC11123948; doi:10.3390/pathogens13050410)
Supplement: Supplementary file 1 [file pathogens-13-00410-s001.zip › pathogens-2972411-supplementary.pdf]

Supplementary material

Table S1 CVB3 specific primers used for PCR fragment preparation and Sanger sequencing

Primer name	Sequence (5’-3’)
C1-S	TAAAACAGCCTGTGGGTTGATC
C1-A	GTAGGTTGATCCATTGGTGG
C2-S	TGGTGTATAATGCAGGCATGG
C2-A	CCCTATAGCGGCTGTTATCG
C3-S	CACTCCTTTCACTTCGCAGG
C3-A	CCCTGTAGTTCCCCACATAC
C4-S	TGTCAACGCTGGAAGCACG
C4-A	CCTCACCACAATTACTAAGGC
C5-S	GGGTCAAGACTCCATCTTAG
C5-A	GTATCCGTCGAAGTGATCTG
C6-S	CAGACCCAGATCACTTCGAC
C6-A	CACCATTGGTAGGTTCAGGC
C7-S	TTGCACTGGGTATCAAGAAGAG
C7-A	AATAATCCGCACCGAATGCG
R_494_513	ACACTGCCCTCTGGCTTGA
F_1045_1064	GTGGGCTATGGAGTATGGC
R_1110_1130	ATGTGGCAACGTCTGGTTGG
R_1937_1959	GATCTCACAGGTATCTGGTATG
F_1961_1982	ATGAAGGATCTGGAACGCAAG
R_ 2897_2919	GAATCAACTTTATCTGGTACAG
F_3543_3565	GTAGAGGTCCAAGAGAGTGAATAC
F_5293_5314	CAAGGTGCTTATACAGGAGTG
F_5824_5844	GGCAAGGTACTGGGTATCC
R_5385_5366	ACGGCGAACTCAAAGGCAG
R_5974_5953	ACTTGGTGTGTTGATGACTGG
F_6867_6887	GGACCAATTCAGGATGATCG
R_6888_6868	GCGATCATCCTGAATTGGTC
